# Monthly Summary

**Published:** 1893-04

**Authors:** 


### Monthly Summary.

MENSTRUATION AND THE TEETH.—In a paper on the "Influence of Menstruation on Incidents of Dental Origin," read before the Odontological Society of Paris, Prof. Sauvez shows that, since the augmentation of the mass of blood during pregnancy is known to produce certain influence on the jaws, that similar conditions existing during menstruation, women are more liable to dental troubles at that period.

On general consideration it would seem to follow that the menstrual outflow, disturbing tbe nervous system and manifesting itself in congestion toward the head and the organs of the chest, would naturally produce a state of least resistance in any tooth susceptible to pulpitis or periostitis.

After observing a large number of cases, the professor sums up his experience as follows:

"In her menstrual period woman is subject to affections of teeth, the same as during pregnancy, which are principally caused by the congestion of the pulp or the periosteum.

"Generally, woman may be said to be disposed to diseases of the teeth when her uterus is troubed.

"The difficulties mentioned occur at the *commencement* of the menstrual period."

The paper concludes with the advice that special care be exercised by practitioners, and that obturation of caries in the fourth degree, for instance, shall not be attempted during the menstrual period.—*Items of Interest.*

HOW TO SLEEP—By Pierre S. Starr, M. D., in *Worthington's Magazine*—Nearly all the methods for inducing sleep are such as tend to withdraw blood from the brain to the skin and abdominal organs. A hot foot bath, for instance, is one of the best of the simple sedatives; a light meal on retiring, or a cracker eaten in the early morning hours when sleep has fled and is wooed in vain, often transfers the seat of activity from the brain to the stomach and brings the former organ into a state conducive to rest. In most cases, however, late suppers should be avoided.

During sleep all the functions of the body are in abeyance, the respiration is slower, the heart beats less rapidly, and digestion is delayed; food, therefore, taken just previous to sleep is apt to be undigested, fermentation results, noxious principles are absorbed, and an unrefreshing, disturbed sleep during the night and dulled faculties in the morning are the result. Light sleepers will generally find a position on the right side the most comfortable and less provocative of unpleasant dreams.

---

A TEMPORARY CROWN—By J. F. Simpson, D. D. S., Trenton, Ont.—Having found that a "Crown *pro tem*" is often very handy while a person is treating up a front tooth that has been broken, I use the following, which a person can make and adjust in ten minutes: Grind broken stump of natural tooth down to near gum border; take an English plate tooth that suits the space, and for a post, a stift piece ot roughened wire flattened at one end; at flattened end, file two small notches in opposite edges, for the pins of the tooth to be bent into to hold the wire firm; this makes the crown, and can be set with gutta percha or "absorbent cotton" wrapped about the wire and put up the root canal; the swelling of the cotton holds it firm in the root, and can be easily removed and reset each time we have to treat the root. Any of my patients who have had to wear this crown have been well satisfied with it. The first one I used was in the case of a gentleman who had just broken off a dead root central. I set him one, and in thirty minutes he was back at work in his store.—*Dominion Dental Journal.*

